# Non-FG mediated transport of the large pre-ribosomal subunit through the nuclear pore complex by the mRNA export factor Gle2

**DOI:** 10.1093/nar/gkt675

**Published:** 2013-07-31

**Authors:** Laura Occhipinti, Yiming Chang, Martin Altvater, Anna M. Menet, Stefan Kemmler, Vikram G. Panse

**Affiliations:** ^1^Department of Biology (D-BIOL), Institute of Biochemistry (IBC), ETH Zurich, Schafmattstrasse 18, CH-8093 Zurich, Switzerland and ^2^MLS Program, Life Sciences Zurich Graduate School, Winterthurerstrasse 190, CH-8057 Zurich, Switzerland

## Abstract

Multiple export receptors passage bound pre-ribosomes through nuclear pore complexes (NPCs) by transiently interacting with the Phe-Gly (FG) meshwork of their transport channels. Here, we reveal how the non-FG interacting yeast mRNA export factor Gly-Leu-FG lethal 2 (Gle2) functions in the export of the large pre-ribosomal subunit (pre-60S). Structure-guided studies uncovered conserved platforms used by Gle2 to export pre-60S: an uncharacterized basic patch required to bind pre-60S, and a second surface that makes non-FG contacts with the nucleoporin Nup116. A basic patch mutant of Gle2 is able to function in mRNA export, but not pre-60S export. Thus, Gle2 provides a distinct interaction platform to transport pre-60S to the cytoplasm. Notably, Gle2’s interaction platforms become crucial for pre-60S export when FG-interacting receptors are either not recruited to pre-60S or are impaired. We propose that large complex cargos rely on non-FG as well as FG-interactions for their efficient translocation through the nuclear pore complex channel.

## INTRODUCTION

A fundamental feature of all eukaryotes is the rapid exchange of diverse macromolecules between the nucleus and cytoplasm. Transport between the two compartments takes place through nuclear pore complexes (NPCs) that are embedded within the double bi-layered nuclear envelope. Shuttling receptors facilitate nucleo-cytoplasmic exchange by binding diverse cargos and simultaneously engaging in transient interactions with Phe-Gly (FG)-rich nucleoporins that line the transport channel of the NPC. Import and export receptors include members of the importin-β-like family, also termed as karyopherins, which recognize either a nuclear localization signal or a nuclear export signal (NES) on cargoes that need to be imported or exported, respectively. In budding yeast, karyopherin-cargo complexes bind to 11 different FG-repeat nucleoporins along the central transport channel (1). The GTPase Ran and its nuclear and cytoplasmic localized regulators (Ran-GAP and Ran-GEF) ensure directionality of karyopherin-mediated transport (2–4).

Pre-ribosomal subunits are amongst the largest (>2.5 MDa) and abundant macromolecular RNA:protein complexes that are assembled in the nucleolus/nucleus, aided by >200 maturation factors (5–7). Export competent pre-ribosomal subunits are exported through the NPC into the cytoplasm by transport factors, where they undergo final maturation steps before initiating translation (8,9). Efficient translocation of a pre-ribosomal particle through the NPC channel presents a major challenge for all eukaryotes (2,10). The essential karyopherin Xpo1 (Crm1 in humans) recognizes leucine-rich NESs found in diverse cargos (11,12) and is required for the export of both pre-60S and pre-40S subunits (13,14). The conserved NES-containing factor Nmd3 is recruited to late pre-60S particles in the nucleus and interacts in a RanGTP-dependent manner with Xpo1 to mediate nuclear export of pre-60S subunits (14,15). Additionally, shuttling *trans*-acting factors such as Arx1, Ecm1, Npl3, Bud20, Rrp12 and Mex67-Mtr2 that can interact directly with the FG-rich hydrophobic meshwork plays a crucial role to facilitate pre-60S subunit nuclear export (16–22). Nevertheless, the interaction networks used to passage a pre-ribosomal subunit through the NPC transport channel remain unresolved.

The conserved NPC associated factor Ribonucleic acid export 1 (Rae1) was initially uncovered in a visual screen in fission yeast aimed at identifying factors involved in mRNA export (23). The budding yeast homolog was subsequently identified in a synthetic lethal genetic screen involving the Gly-Leu-FG (GLFG) nucleoporin Nup100, and hence it was termed GLFG lethal 2 (Gle2) (24). The mammalian homolog of Rae1 was independently isolated from sub-fractions of rat liver nuclear envelopes (25). Gle2 is tethered to the NPCs, not via interactions with FG-repeats (26) but rather by a conserved non-FG interaction involving a 57-residue stretch within the N-terminal region of GLFG nucleoporin Nup116 (Nup98 in mammals) termed the Gle2-binding sequence (GLEBS) (27,28).

Structural analysis of the human Rae1:Nup98-GLEBS complex revealed that Rae1 belongs to the WD40 domain protein family that forms a seven-bladed β-propeller with several extensive surface loops (29). A long hairpin of the Nup98-GLEBS motif binds an invariant hydrophobic surface of Rae1 that extends over the top of its WD40 propeller. The Rae1:Nup98-GLEBS complex surface features a conserved basic patch located along one side of the Rae1 WD40 propeller. Biochemical studies showed that the Rae1:Nup98-GLEBS complex can bind ssRNA *in vitro*. Whether the basic patch contributes to ssRNA binding *in vitro* and mRNA export *in vivo*, however, was not investigated (29).

Rae1 has been implicated in the mRNA export pathway, as the inactivation of Rae1 in fission yeast induces nuclear accumulation of poly-(A)^+^ RNA (23). Interestingly, depletion of Rae1 does not induce an mRNA export defect in mice or in cultured *Drosophila* SL2 cells (30). However, overexpression of the GLEBS motif of Nup98 causes mislocalization of Rae1 and induces nuclear accumulation of poly-(A)^+^ RNA in HeLa cells, arguing for a role of Rae1 in the mammalian mRNA export pathway (28). In budding yeast, Gle2 is crucial for nuclear export of poly-(A)^+^RNA under various stress conditions, including heat shock and treatment with ethanol or benzyl alcohol (31). Several viral proteins, including the matrix (M) protein of the vesicular stomatitis virus and the NS1 protein of influenza virus, inhibit nuclear export of host mRNAs and host cell transcription by directly targeting Nup98 and Rae1, underscoring the importance of the interaction between Rae1 and Nup98 to regulate gene expression (32–34).

In addition to its role in mRNA export, Rae1 influences multiple aspects of the cell cycle. Rae1 was identified as an essential mitotic checkpoint regulator that prevents chromosome missegregation (35). Rae1 also stabilizes cytoplasmic microtubules, regulating the organization of the cytoskeletal network during mitosis (36). Rae1 was shown to interact with the nuclear mitotic apparatus as well as the cohesion subunit Smc1 to direct proper spindle formation and influence chromosome segregation (37,38). The Rae1:Nup98 complex was also identified as a temporal regulator of Cdh1-activated anaphase-promoting complex and acts to inhibit Securin degradation and prevent aneuploidy (39,40). More recently, Rae1 was identified as a functional component of the evolutionarily conserved Highwire (Hiw) Fsn E3 ubiquitin ligase complex that acts by preventing autophagy-mediated degradation of the Hiw protein to regulate synaptic development in post-mitotic neurons in *Drosophila* and *Caenorhabditis elegans* (41,42).

In this study, we reveal an adjunct role for Gle2 in pre-60S transport. To bind pre-60S, Gle2 uses an exposed basic patch. This interaction is specific for pre-60S, as mutating this surface affects their export but does not impair mRNA export. We also find a second surface used by Gle2 for pre-60S subunit export that engages in non-FG contacts with the nucleoporin Nup116. Both interaction surfaces of Gle2 become crucial *in vivo* for pre-60S export when FG-interacting transport receptors are either impaired or limiting. Our findings suggest that large complex cargos rely on multiple non-FG as well as FG interactions for their efficient translocation through the NPC channel.

## MATERIALS AND METHODS

### Yeast strains and plasmids

The *Saccharomyces cerevisiae* strains used in this study are presented in Supplementary Table S1. All genetic manipulations were performed as previously described (43,44). Preparation of media, yeast transformation, mating, diploid sporulation, tetrad analysis and genetic manipulation were performed according to established protocols (45). Plasmids used in this study are listed in Supplementary Table S2. All recombinant DNA techniques were performed according to established procedures using *Escherichia coli* XL1 blue cells for cloning and plasmid propagation. All cloned DNA fragments generated by PCR amplification were verified by sequencing. Site-directed mutagenesis was performed by QuikChange XL kit (Agilent Technologies, Switzerland) according to the instructions. Mutagenized plasmids were verified by sequencing.

### Genetic analyses

All genetic analyses reported in this study were performed as previously described (46).

### Tandem affinity purifications and western analyses

Whole-cell extracts were prepared as previously described (47). Tandem affinity purifications (TAP) of pre-ribosomal particles were carried out as previously described (18,47). Eluates were analyzed in NuPAGE 4–12% Bis-Tris gels (Invitrogen, Switzerland) followed by silver staining or western analyses.

Western analyses were performed as previously described (47). The following primary antibodies were used in this study: α-Bud20 (1:2000; this study), α-GFP (1:2000; Roche, Switzerland), α-Gle2 (1:500; this study), α-Mex67 (1:5000; C Dargemont, Institut Jacques Monod, Paris, France), α-Nmd3 (1:5000; A Johnson, University of Texas at Austin, Austin TX, USA), α-Noc1 (1:1000; H Tschochner, Universität Regensburg, Regensburg, Germany), α-Nog1 (1:2000; M Fromont-Racine, Institut Pasteur, Paris, France), α-Nsa2 (1:2000; M Fromont-Racine, Institut Pasteur, Paris, France), α-Nug1 (1:2000; this study), α-Rlp24 (1:2000; M Fromont-Racine, Institut Pasteur, Paris, France), α-Rpl35 (1:4000; this study), α-TAP (1:5000, Thermo Scientific, Rockford, IL, USA), α-Tif6 (1:2000; GenWay Biotech, USA) and α-Yvh1 (1:4000; this study). The secondary horseradish peroxidase-conjugated α-rabbit, α-mouse and α-chicken antibodies (Sigma-Aldrich, USA) were used at 1:1000–1:5000 dilutions. Protein signals were visualized using Immun-Star horseradish peroxidase chemiluminescence kit (Bio-Rad Laboratories, USA) and captured by Fuji Super RX X-ray films (Fujifilm, Japan).

### Polysome analysis

Sedimentation analysis of yeast lysates by sucrose gradient ultracentrifugation was performed as described previously (47). Briefly, the indicated strains were grown to OD_600_ = 0.8 before treatment with 100 µg/ml cycloheximide. The culture was cooled on ice for 5 min. Cells were harvested and washed once in lysis buffer [10 mM Tris–Cl (pH 7.4), 100 mM NaCl, 30 mM MgCl_2_ and 100 µg/ml cycloheximide] and disrupted by glass bead lysis in 1 ml of lysis buffer. After clarification of the lysate (14 000 rpm for 10 min at 4°C), the supernatant corresponding to 4 OD_260_ units was loaded onto a 7–50% sucrose density gradient containing 50 mM Tris–acetate (pH 7.4), 50 mM NH_4_Cl and 12 mM MgCl_2_. The gradient was centrifuged at 39 000 rpm for 165 min using a SW41 rotor (Beckman Coulter, USA) and analyzed at 254 nm using a density gradient fractionator.

### Fluorescence *in situ* hybridization

Nuclear accumulation of poly-(A)^+^ RNA was monitored by fluorescence *in situ* hybridization (48) using a Cy3-oligo (dT)^30^ probe as previously described (46).

### Fluorescence microscopy

Cells were visualized using DM6000B microscope (Leica, Germany) equipped with HCX PL Fluotar 63×/1.25 NA oil immersion objective (Leica, Germany). Images were acquired with a fitted digital camera (ORCA-ER; Hamamatsu Photonics, Japan) and Openlab software (Perkin-Elmer, USA).

## RESULTS

### Gle2 genetically interacts with pre-60S subunit export receptors

**Figure 1.**
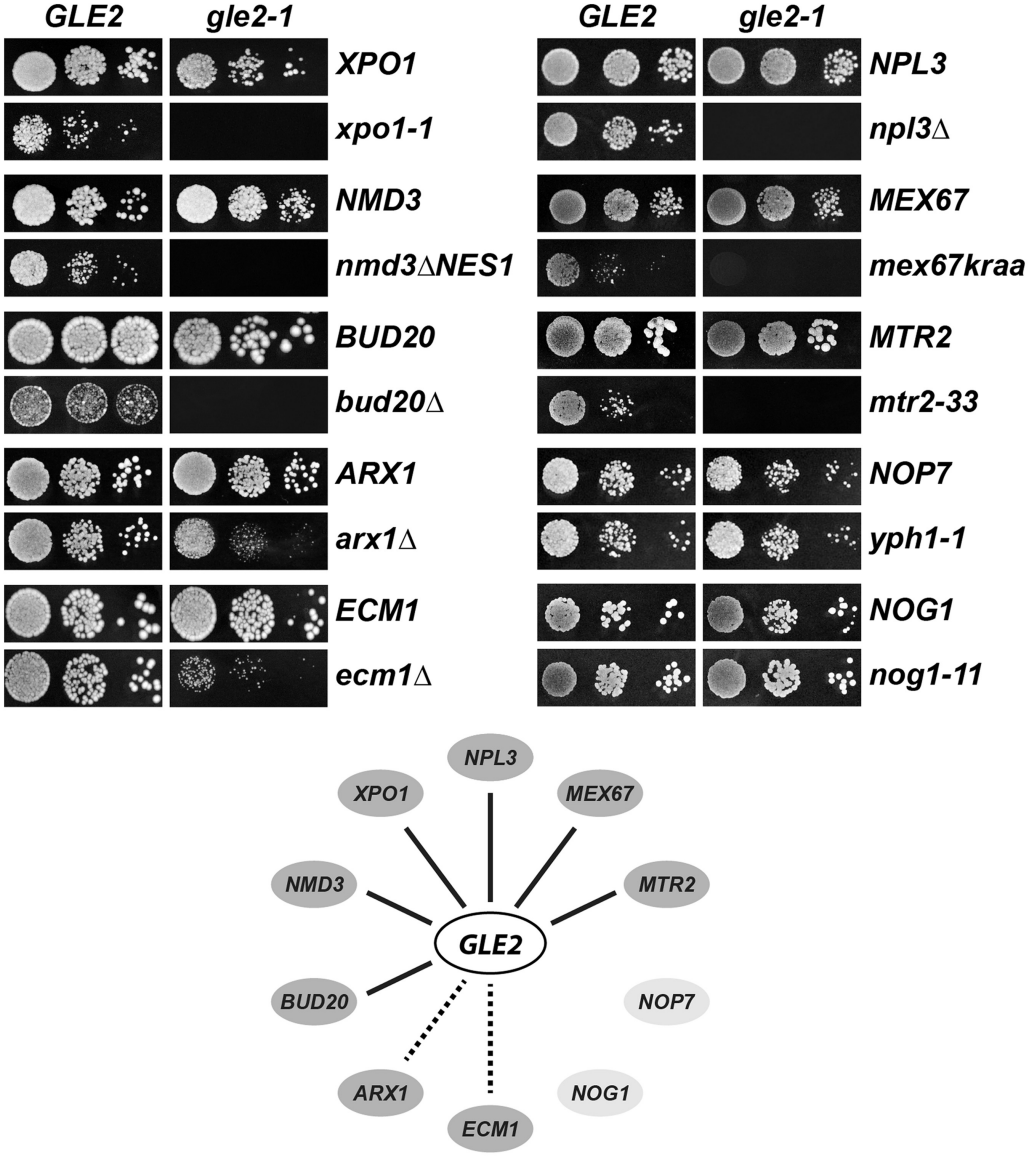
Gle2 genetically interacts with factors required for proper pre-60S subunit export. The *gle2-1* allele is synthetic lethal (*sl*, solid line) or synthetic enhanced (*se*, dashed line) when combined with impaired alleles or deletions of factors required for pre-60S subunit export. *gle2-1* allele is *sl* with *xpo1-1*, *nmd3ΔNES1*, *bud20Δ*, *npl3Δ*, *mex67kraa* and *mtr2-33* mutants and is strongly *se* when combined with *arx1Δ* and *ecm1Δ* mutants. *gle2-1* allele is not *sl* or *se* when combined with the indicated *yph1-1* and *nog1-11* mutants. Strains carrying the WT or mutant alleles were spotted in 10-fold serial dilutions on synthetic defined (SD) plates containing 5-Fluoroorotic Acid (5-FOA, when *sl*) or yeast extract peptone dextrose (YPD) plates (when *se* or no interaction) and grown at 25–30°C for 3–5 days. Factors that function in pre-60S nuclear export are indicated in dark gray, pre-60S associated assembly factors are indicated in light gray.

Gle2 was reported to interact genetically with the non-karyopherin pre-60S subunit export receptor Arx1 (16,22,49,50). These studies prompted us to explore whether Gle2 plays a direct role in pre-60S subunit export. We began our investigation by testing whether Gle2 genetically interacts with other factors that directly participate in pre-60S subunit export. For our analyses, we used the *gle2-1* allele that contains a premature stop codon at position W334, which results in a truncated and unstable protein, and renders the *gle2-1* strain a temperature sensitive growth phenotype (24). Consistent with a role for Gle2 in pre-60S subunit nuclear export, the *gle2-1* strain exhibited synthetic lethal (e.g. *xpo1-1*, *nmd3ΔNES1*, *bud20Δ, npl3Δ*) or strong synthetic enhanced (*arx1Δ* and *ecm1Δ*) growth defects when combined with mutants of known pre-60S subunit export factors (Figure 1). Notably, alleles of the general mRNA export factors Mex67-Mtr2 (*mex67kraa* and *mtr2-33*) that are specifically impaired in the nuclear export of pre-60S subunits, but not mRNA export, were synthetically lethal with *gle2-1* (Figure 1). In contrast, mutant alleles of the nucleolar/nuclear pre-60S associated maturation factor Nop7 (*yph1-1*) (51) and the shuttling GTPase Nog1 (*nog1-11*) (52) showed no synthetic interaction with *gle2-1* (Figure 1). These genetic studies suggest that Gle2 functionally overlaps with FG-interacting pre-60S export receptors, but not with pre-60S subunit associated maturation factors.

### A role for Gle2 in the nuclear export of pre-60S subunits

**Figure 2.**
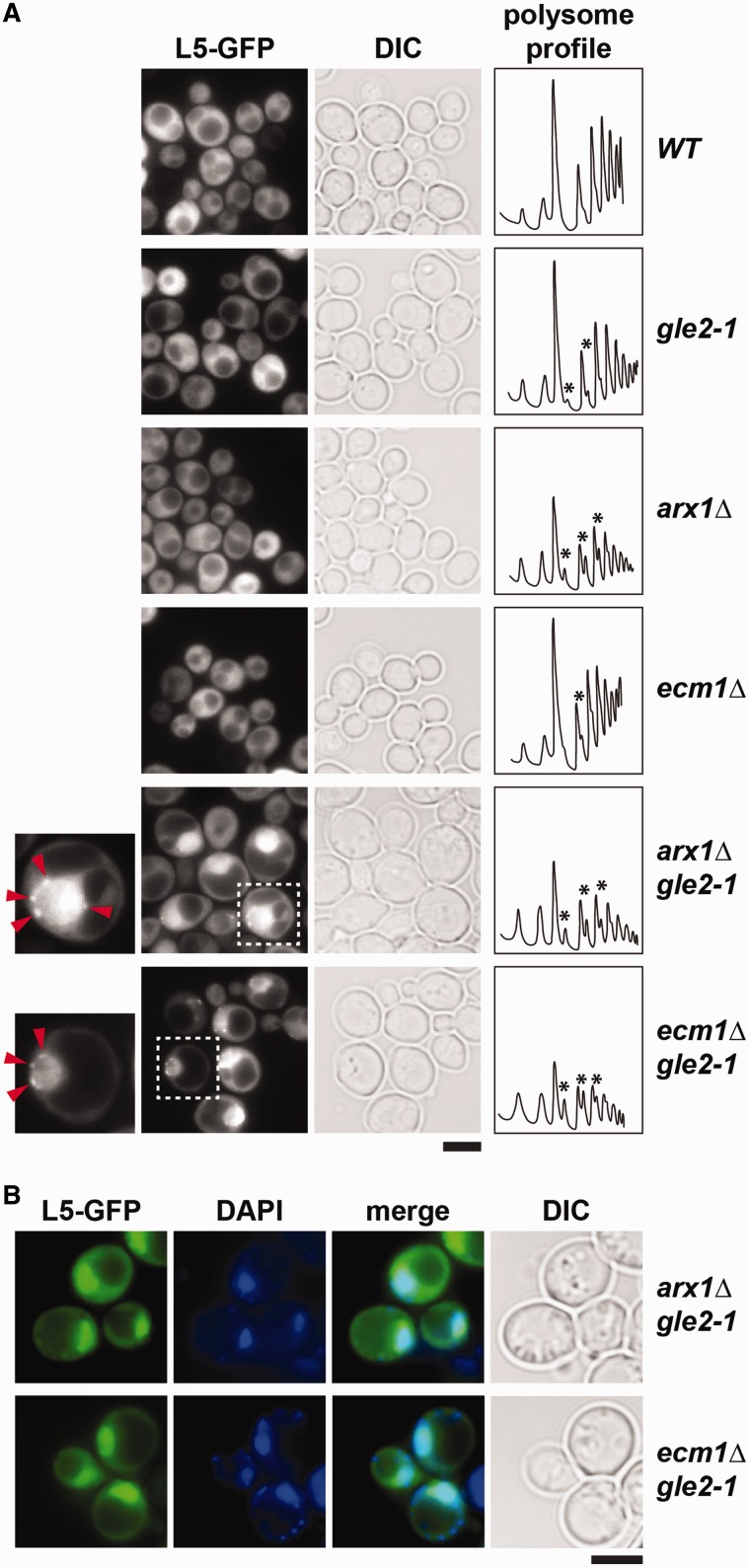
A role for Gle2 in the nuclear export of pre-60S subunits. (**A**) The *arx1Δgle2-1* and *ecm1Δgle2-1* double mutants are impaired in pre-60S subunit export. Indicated strains containing L5-GFP reporter were grown at 30°C till mid log phase. Localization of L5-GFP reporter was monitored by fluorescence microscopy. The insets on the left are magnified images of the punctate staining of L5-GFP (red arrows) in *arx1Δgle2-1* and *ecm1Δgle2-1* double mutant strains. Polysome analyses of cell lysates derived from the indicated yeast strains by sucrose density gradient sedimentation. The strains were grown at 30°C till mid log phase and treated with 100 µg/ml cycloheximide to preserve polysomes. In all, 4 OD_260_ units of the clarified lysates were fractionated by 7–50% sucrose density ultracentrifugation and analyzed at 254 nm using a density gradient fractionator. Halfmers in the depicted profiles are indicated with asterisks. (**B**) The pre-60S subunit reporter L5-GFP co-localizes with the nuclear marker DAPI in *arx1Δgle2-1* and *ecm1Δgle2-1* double mutant strains. Indicated strains containing L5-GFP were grown at 30°C till mid log phase, incubated with 10 µg/ml DAPI for 30 min before inspection by fluorescence microscopy. Scale bar = 5 µm.

Next, we investigated whether Gle2 plays a specific role in the transport of pre-60S subunits to the cytoplasm. For this, we monitored nuclear export of the pre-60S subunits using the large subunit reporter L5-GFP in the synthetically enhanced *arx1Δgle2-1* and *ecm1Δgle2-1* mutant strains. Although the single mutant strains *gle2-1*, *arx1Δ* and *ecm1Δ* did not show any apparent pre-60S subunit export defect, the double mutant strains *arx1Δgle2-1* and *ecm1Δgle2-1* exhibited strong nucleoplasmic accumulation of L5-GFP at 30°C, as judged by the co-localization with the nuclear marker DAPI (Figure 2A and B). Often, a punctate accumulation of the L5-GFP reporter at the nuclear periphery was observed in these double mutant strains (insets in Figure 2A).

Next, we analyzed the lysates from the single mutant (*gle2-1*, *arx1Δ* and *ecm1Δ*) and the double mutants (*arx1Δgle2-1* and *ecm1Δgle2-1*) by sucrose gradient sedimentation. These analyses revealed presence of halfmers in the polysome profiles of the *gle2-1* and *arx1Δ* mutants, indicating a deficit of cytoplasmic 60S subunits. This halfmer phenotype was found to be weaker in the *ecm1Δ* mutant. Consistent with the strong nuclear accumulation of L5-GFP, the polysome profiles of the *arx1Δgle2-1* and *ecm1Δgle2-1* mutants show a significant decrease in the 80S peak, as compared with the single mutants, as well as presence of halfmers. Together, these data indicate a strong deficit in cytoplasmic 60S subunits in the double mutants (Figure 2A).

At restrictive temperature (37°C), the *gle2-1* mutant exhibits a ‘pore-clustering’ phenotype, similar to that observed in the *nup133Δ* mutant (Supplementary Figure S1A) (24). To determine whether the observed L5-GFP localization defect was a result of NPC clustering, as seen in *nup133Δ* cells (Supplementary Figure S1A), we investigated the NPC distribution in the *arx1Δgle2-1* and *ecm1Δgle2-1* strains. At 30°C, when both strains are impaired in pre-60S subunit export, the punctate nuclear rim localization of the nucleoporin Nup49-GFP was indistinguishable from WT and single mutants, indicating that NPC distribution is not affected in these mutants (Supplementary Figure S1A). Moreover, nuclear export of pre-40S subunits and mRNAs remained unaffected as monitored by the *in vivo* localization of the small subunit reporter S2-GFP and poly-(A)^+^ RNA, respectively (Supplementary Figure S1B). Thus, the strong growth defects observed in the *arx1Δgle2-1* and *ecm1Δgle2-1* mutants correlate with impaired pre-60S subunit export. Based on these data, we conclude that Gle2 becomes crucial for nuclear export of pre-60S subunits in the absence of or impairment in any one of its export receptors.

### Gle2 co-enriches with late pre-60S subunits

**Figure 3.**
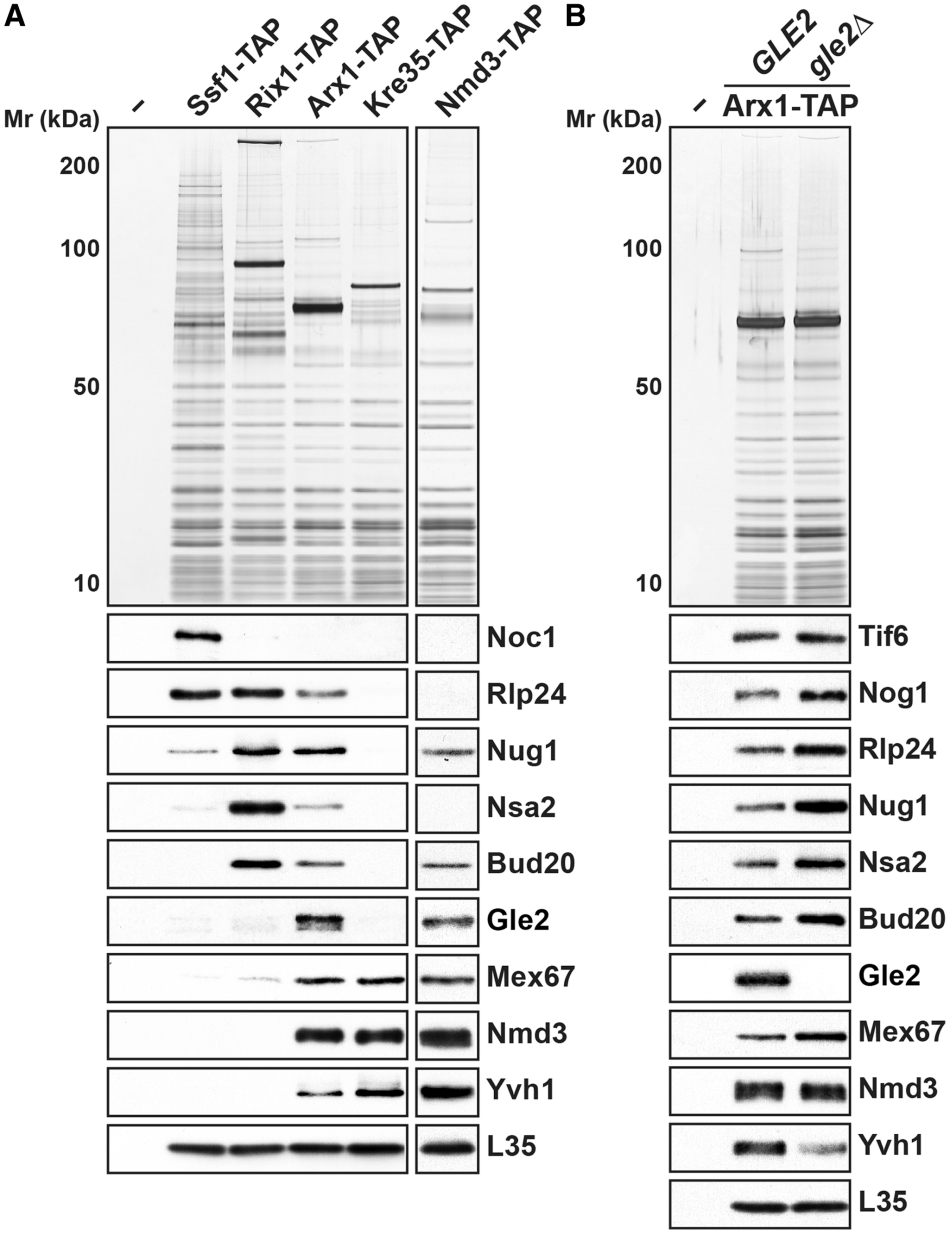
Gle2 co-enriches with only late export competent pre-60S subunits. (**A**) Gle2 co-enriches with late pre-60S subunits. TAP of pre-60S subunits was performed *via* the indicated TAP-tagged bait proteins. (**B**) TAP purifications using Arx1 as bait were performed in WT *(GLE2)* and *gle2Δ* strains. (A and B) The calmodulin-sepharose eluates were analyzed on NuPAGE 4–12% gradient gels followed by silver staining and western analyses. The large subunit ribosomal protein L35 served as loading control.

The observations that Gle2 strongly genetically interacts with pre-60S subunit export receptors and that the *gle2-1* mutant exacerbates the pre-60 S subunit export defect when combined with *arx1Δ* and *ecm1Δ* mutants led us to investigate whether Gle2 binds pre-60 S subunits *in vivo*. To this end, we isolated the well characterized early to late pre-60 S particles using different TAP-tagged bait proteins: Ssf1 (early nucleolar), Rix1 (nucleoplasmic), Arx1 (late export competent), and Kre35 (cytoplasmic) (18,53,54). Western analyses were performed to verify the isolated pre-60S particles using antibodies directed against several *trans*-acting and transport factors: the early nucleolar [intra-nuclear transport factor Noc1 (55)], late nucleolar/early nucleoplasmic [ribosomal-like protein Rlp24 (56,57), the shuttling GTPase Nug1 (58), shuttling *trans*-acting factor Nsa2 (59,60), and the FG-interacting pre-60 S subunit export receptor Bud20 (18,21)], late nucleoplasmic [export factors Mex67 and Nmd3 (13,54)] and cytoplasmic [Yvh1, the cytoplasmic release factor for the shuttling ribosomal-like protein Mrt4 (47,61)] pre-60 S particles. To detect Gle2 in our purifications, we tagged Gle2 with several epitopes (-FLAG, -HA, -*myc* and -GFP) at both N- and C-termini. However, the various constructs were either strongly synthetically enhanced or synthetically lethal when combined with different pre-60 S export factor mutants (e.g. *xpo1-1*, *nmd3ΔNES1*, *mex67kraa* and *mtr2-33*), suggesting that the tagged versions of Gle2 are non-functional in pre-60S subunit export (data not shown). Hence, co-enrichment with different pre-60S particles was assessed with antibodies generated against Gle2. These analyses showed that Gle2 co-enriched only with the export competent pre-60S particles, isolated using the shuttling bait Arx1-TAP that also contained export factors Nmd3, Bud20 and Mex67-Mtr2 (Figure 3A), but not with the early (Ssf1-TAP and Rix1-TAP) as well as late cytoplasmic (Kre35-TAP) 60S pre-ribosomes. The western signal for Gle2 was specific, as it was lost when Arx1-TAP was isolated from the *gle2Δ* mutant (Figure 3B). Consistent with this, Gle2 co-enriches with another late pre-60S subunit isolated *via* Nmd3-TAP, the NES containing adaptor for the export receptor Xpo1 (Figure 3A). Based on these data, we conclude that Gle2 co-enriches with late pre-60S particles that are loaded with other export receptors.

Notably, a modest increase in levels of shuttling *trans*-acting factors (Tif6, Nog1, Nug1, Rlp24, Nsa2 and Bud20) and the export factor Mex67 is seen in Arx1-TAP isolated from the *gle2Δ* mutant (Figure 3B). Moreover, a reduction in the levels of Yvh1, a factor that joins pre-60S particles in the cytoplasm (Figure 3B), was seen in this purification. These data show that Gle2 is not involved in the recruitment of export factors, as Nmd3, Mex67-Mtr2 and Bud20 were still bound to Arx1-TAP in the strain lacking Gle2 (Figure 3B). Western analysis of whole-cell lysates derived from Arx1-TAP and Arx1-TAP *gle2Δ* strains show that the Arx1-TAP is present at similar levels in both strains (Figure 6A, bottom), suggesting the absence of Gle2 did not alter the protein levels of Arx1-TAP. Based on all the aforementioned genetic, cell-biological and biochemical data, we suggest that Gle2-deficient cells are delayed in pre-60S subunit nuclear export that is not apparent from the localization of the large subunit reporter L5-GFP (Figure 2A).

### A role for the conserved exposed basic patch of Gle2 in pre-60S subunit binding and nuclear export

**Figure 4.**
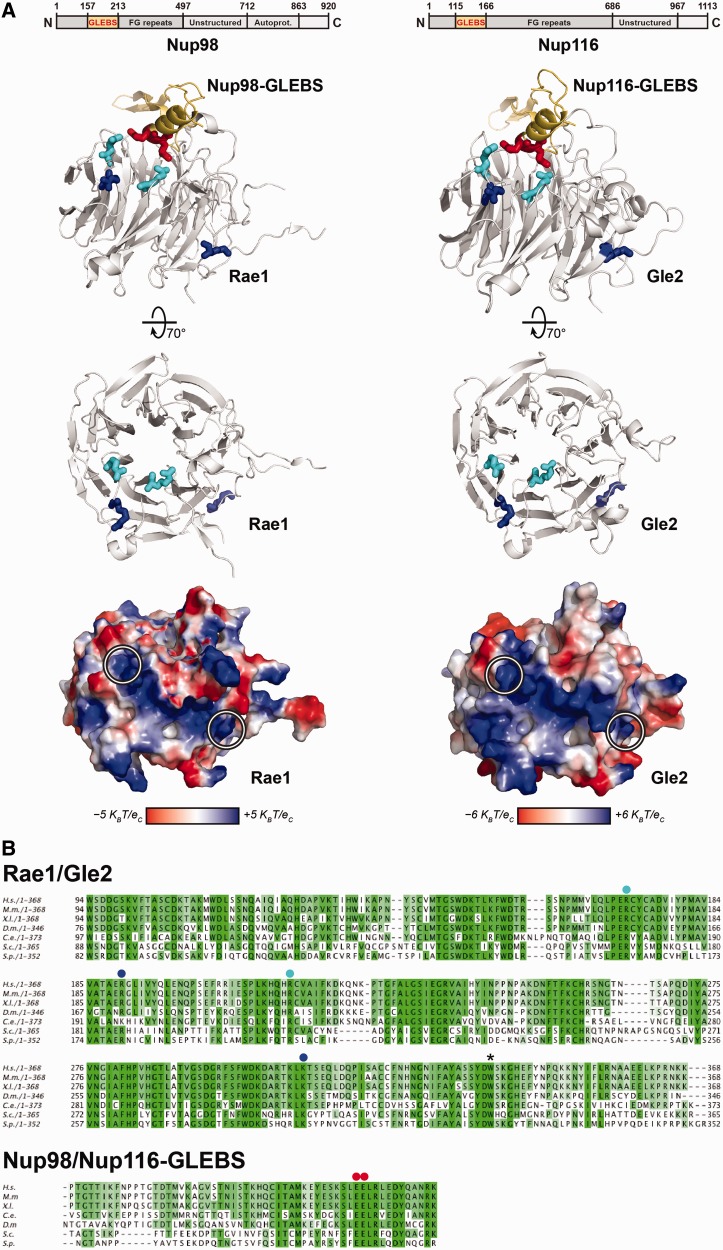
Conserved interactions of the Rae1:Nup98-GLEBS crystal structure and the modeled Gle2:Nup116-GLEBS interface. (**A**) Top: Schematic representation of Nup98 and Nup116 domain organization. Residue numbers indicate the domain boundaries. Center: Ribbon representations of Rae1:Nup98-GLEBS (left) and Gle2:Nup116-GLEBS (right) complexes, visualized by PyMOL. Structure information of Rae1:Nup98-GLEBS was acquired from the Protein Data Bank (DOI:10.2210/pdb3mmy/pdb) (29). The structure modeling of Gle2:Nup116-GLEBS was performed by using PHYRE2 server (62). Of the 365 residues of Gle2, 339 residues, i.e. 93% of Gle2 primary sequence, could be modeled with high confidence using Rae1:Nup98-GLEBS as template. Conserved residues in the basic patch are indicated in blue. Conserved residues involved in making salt bridges in Rae1:Nup98-GLEBS or potentially involved in making salt bridges in Gle2:Nup116-GLEBS are indicated in cyan (in Rae1/Gle2) and red (in Nup98/Nup116). Bottom: Van der Waals surface representation of Rae1 and Gle2. The two conserved arginine and lysine residues located at the ends of the conserved basic patch are highlighted with white circles. *K_B_*, Boltzmann’s constant (1.381 × 10^−23^ JK^−1^); *T*, temperature (310 K); *e_c_*, elementary charge (1.602 × 10^−19^ C). (**B**) Top: Sequence alignment of Rae1 (partial) from *Homo sapiens* (*H.s.*), *Mus musculus* (*M.m.*), *Xenopus laevis* (*X.l.*), *Drosophila melanogaster* (*D.m.*), *Caenorhabditis elegans* (*C.e.*), *Saccharomyces cerevisiae* (*S.c.*) and *Schizosaccaromyces pombe* (*S.p.*) using ClustalW program (63). Bottom: Sequence alignment of the GLEBS domain of human Nup98 with its homologs from various organisms using ClustalW program. The overall sequence conservation at each position is shaded in a color gradient from light green (40% identity) to dark green (100% identity). Conserved residues (R186 and K305 in *S.c.* Gle2) that were mutated in *gle2-patch* allele are marked with blue dots. Conserved residues (R168 and R212 in *S.c.* Gle2) that were mutated in *gle2-gis* mutant are marked with cyan dots. Conserved residues (E154 and E155 in *S.c.* Nup116) of the Nup98-GLEBS that are involved in making salt bridges with Rae1 are marked with red dots. The asterisk indicates the conserved tryptophan mutated to a stop codon in the *gle2-1* allele.

**Figure 5.**
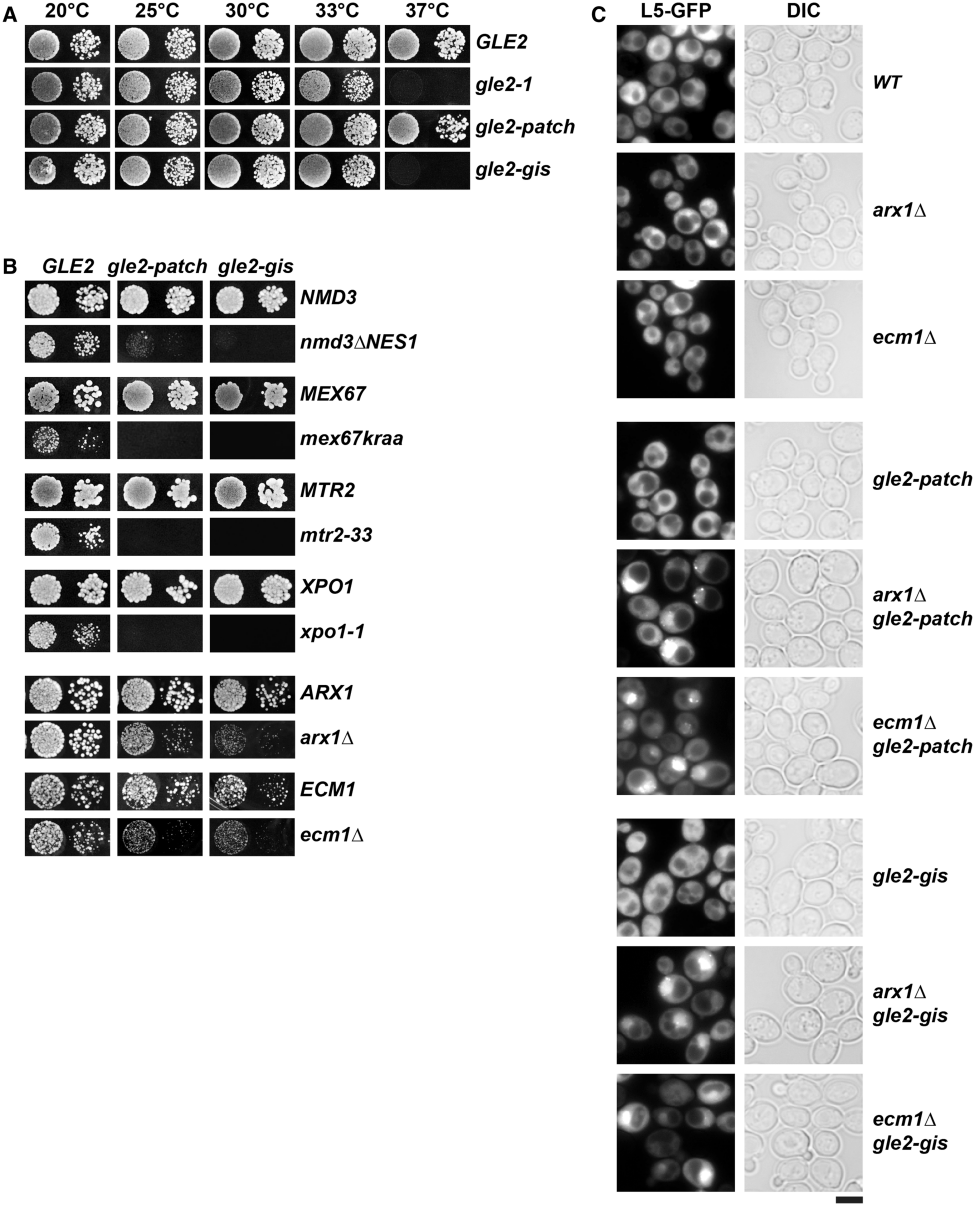
The conserved basic patch and GLEBS-interacting surface contributes to Gle2 function in pre-60S subunit nuclear export. (**A**) The *gle2-1* and *gle2-gis* mutant strains are temperature sensitive *(ts*) at 37°C. WT (*GLE2*), *gle2-1*, *gle2-patch* and *gle2-gis* strains were spotted in 10-fold serial dilutions on YPD plates and grown at indicated temperatures for 2–4 days. (**B**) The *gle2-patch* and *gle2-gis* mutants genetically interact with pre-60S export factors. Both alleles are *sl* when combined with the *nmd3ΔNES1*, *mex67kraa, mtr2-33* and *xpo1-1* mutants and strongly *se* when combined with *arx1Δ* and *ecm1Δ* mutants. Strains carrying the WT and mutant alleles were spotted in 10-fold serial dilutions on 5-FOA (SD) plates and grown at 25–30°C for 3–5 days. (**C**) The *arx1Δgle2-patch, ecm1Δgle2-patch, arx1Δgle2-gis* and *ecm1Δgle2-gis* strains are impaired in pre-60S subunit export. The indicated strains containing L5-GFP reporter were grown at 30°C till mid log phase. Localization of L5-GFP reporter was analyzed by fluorescence microscopy. Scale bar = 5 µm.

Structural analysis of the human Rae1:Nup98-GLEBS complex identified two surfaces present on the WD40 fold of Rae1 (29). The first conserved GLEBS-interacting surface is present on top of the WD40 fold that anchors Rae1 to the NPC *via* non-FG interactions with the GLEBS motif present within the nucleoporin Nup98. A second yet uncharacterized exposed conserved basic patch is juxtaposed adjacent to the GLEBS-interacting surface and runs along the circumference of the WD40 fold (Figure 4A). We tested whether the equivalent basic patch on yeast Gle2 plays a role in the pre-60S subunit export. As previously noted, the homology model of Gle2:Nup116-GLEBS generated using the Rae1:Nup98 complex as template displayed the conserved basic patch along the circumference of the β-propeller (Figure 4A). We mutated the two conserved basic residues (R186 and K305, indicated with blue dots in Figure 4B) located at the ends of the basic patch on Gle2 (indicated with blue side chains and white circles in Figure 4A) to generate the *gle2-R186E*,*K305E* allele, hereafter termed as ‘*gle2-patch*’ allele. Unlike the *gle2-1* allele (24), the *gle2-patch* mutant strain was not temperature sensitive and grew nearly at the WT rate in the temperature range of 20–37°C (Figure 5A).

The contribution of these conserved basic residues to Gle2 function in pre-60S subunit nuclear export was assessed using genetic and cell-biological approaches. Like the *gle2-1* allele, the *gle2-patch* mutant was synthetically lethal when combined with pre-60S subunit export mutants (*nmd3ΔNES1*, *mex67kraa*, *mtr2-33*, *bud20Δ* and *xpo1-1*) and induced strong synthetic growth defects when combined with *arx1Δ* or *ecm1Δ* (Figure 5B). Next, we examined the localization of the large subunit reporter L5-GFP in the synthetically enhanced *arx1Δgle2-patch* and *ecm1Δgle2-patch* mutant strains. As expected, these double mutant strains exhibited a strong nuclear accumulation of L5-GFP, indicating impairment in pre-60S subunit export (Figure 5C). We suggest that the basic patch contributes to the function of Gle2 in pre-60S subunit nuclear export.

**Figure 6.**
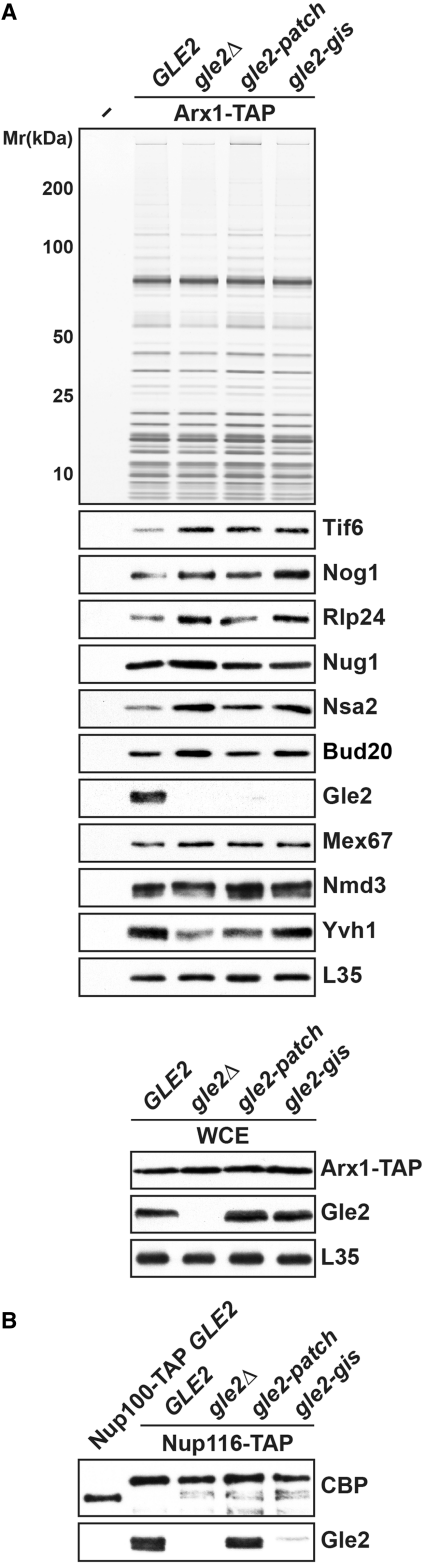
Recruitment of Gle2 to late pre-60S subunits requires its basic patch and the GLEBS-interacting surface. (**A**) Top: *gle2-patch* and *gle2-gis* mutant proteins do not co-enrich with export competent pre-60S subunits. TAP purifications using Arx1 as bait were performed in WT (*GLE2*), *gle2Δ*, *gle2-patch* and *gle2-gis* mutant strains. The calmodulin-sepharose eluates were analyzed on NuPAGE 4–12% gradient gels followed by silver staining and western analyses. The large subunit ribosomal protein L35 served as loading control. Bottom: *gle2-patch* and *gle2-gis* mutant proteins are expressed at WT level. The levels of Gle2, *gle2-patch* and *gle2-gis* proteins were examined in whole-cell extracts (WCE) by western analysis. (**B**) The GLEBS-interacting surface of Gle2 is required for anchoring to the NPC. TAP purifications were performed using Nup100 and Nup116 as bait proteins. The tobacco etch viral (TEV) protease treated eluates were examined by western analyses using α-TAP (CBP) and α-Gle2 antibodies.

The aforementioned data prompted us to test whether the Gle2 basic patch is required to bind pre-60S subunits. We isolated Arx1-TAP from strains containing WT *GLE2* and the *gle2-patch* mutant. Although WT Gle2 efficiently co-enriched with Arx1-TAP, the *gle2-patch* mutant protein failed to co-enrich with Arx1-TAP (Figure 6A, top). This lack of co-enrichment was not due to a destabilized mutant protein, as western analyses of whole-cell extracts revealed that the *gle2-patch* mutant protein was expressed at level similar to WT Figure 6A, bottom). As the conserved basic patch is juxtaposed adjacent to the GLEBS-interacting surface of Gle2, we investigated whether mutations in the basic patch of Gle2 perturb interactions with the GLEBS motif within Nup116. To this end, we isolated Nup116-TAP from WT and *gle2-patch* mutant strains. The *gle2-patch* mutant protein co-enriched with Nup116-TAP as efficiently as WT Gle2 (Figure 6B).

Together, these data show that mutations within the conserved basic patch of Gle2 affect pre-60S binding, but interactions with the nucleoporin Nup116 remain unaltered.

### A role for the GLEBS-interacting surface of Gle2 in pre-60S subunit binding and nuclear export

Structural analysis of the Rae1:Nup98-GLEBS complex revealed the precise molecular interactions on the top surface of the β-propeller that make non-FG contacts with the GLEBS motif in the N-terminal region of Nup98 (Figure 4A, left). This analysis showed that the Rae1:Nup98-GLEBS interface involves several side-chain interactions, including two conserved salt bridges between R216 and R172 of Rae1 and E201 and E202 of the GLEBS motif of Nup98 (depicted with cyan and red residues in Figure 4A, left), that play a crucial role in the Rae1:Nup98-GLEBS interaction and are highly conserved from yeast to humans (Figure 4B) (29). We investigated the contribution of the equivalent non-FG Gle2:Nup116-GLEBS interaction to pre-60S subunit export. Sequence alignments identified two basic residues (R168 and R212) of Gle2 (depicted with cyan residues and dots in Figure 4A, right and B) that potentially could make salt bridges with glutamate residues (E154 and E155) on the Nup116-GLEBS motif (depicted with red residues and dots in Figure 4A, right and B). We mutated R168 and R212 within Gle2 to glutamates to generate the *gle2-R168E,R212E* allele, hereafter termed as ‘*gle2-gis*’ (*gle2-glebs interacting surface*) allele. Similar to the *gle2-1* allele, we found that the *gle2-gis* mutant was unable to grow at 37°C (Figure 5A).

We investigated the interaction of the *gle2-gis* mutant protein with the nucleoporin Nup116. To this end, Nup116-TAP was isolated from WT and *gle2-gis* strains, and co-enrichment of the WT and mutant protein was assessed using antibodies directed against Gle2. As controls, we isolated Nup116-TAP from the *gle2Δ* mutant and Nup100-TAP, another functionally overlapping GLFG nucleoporin that lacks a GLEBS motif. In agreement with previous studies, we found that Gle2 efficiently co-enriched with Nup116-TAP, but not with Nup100-TAP (Figure 6B). The western signal was specific, as it was not present in Nup116-TAP complexes isolated from the *gle2Δ* strain (Figure 6B). Consistent with the conserved role of salt bridges in stabilizing Nup116:Gle2 interactions, we found that the *gle2-gis* mutant protein failed to efficiently co-enrich with Nup116-TAP (Figure 6B).

We assessed the contribution of Gle2’s GLEBS-interacting surface to pre-60S subunit export using genetic and cell-biological approaches. Like the *gle2-1* and *gle2-patch* alleles, the *gle2-gis* mutant was synthetically lethal when combined with the pre-60S subunit export mutants (*nmd3ΔNES1*, *mex67kraa*, *mtr2-33, bud20Δ* and *xpo1-1*) and induced strong synthetic growth defects when combined with *arx1Δ* or *ecm1Δ* (Figure 5B). Moreover, the synthetically enhanced *arx1Δgle2-gis* and *ecm1Δgle2-gis* mutants exhibited a strong nuclear accumulation of the large subunit reporter L5-GFP, indicating impairment in pre-60S subunit export (Figure 5C).

Next, we investigated whether the GLEBS-interacting surface of Gle2 also contributes to pre-60S subunit recruitment. For this, we isolated Arx1-TAP from WT and *gle2-gis* mutant strains. Although Gle2 efficiently co-enriched with Arx1-TAP, the *gle2-gis* mutant protein failed to co-enrich with Arx1-TAP (Figure 6A, top). This lack of co-enrichment with both Nup116-TAP and Arx1-TAP was not due a destabilized mutant protein, as western analysis of whole-cell lysates revealed that the *gle2-gis* mutant protein was expressed at level similar to WT (Figure 6A, bottom).

**Figure 7.**
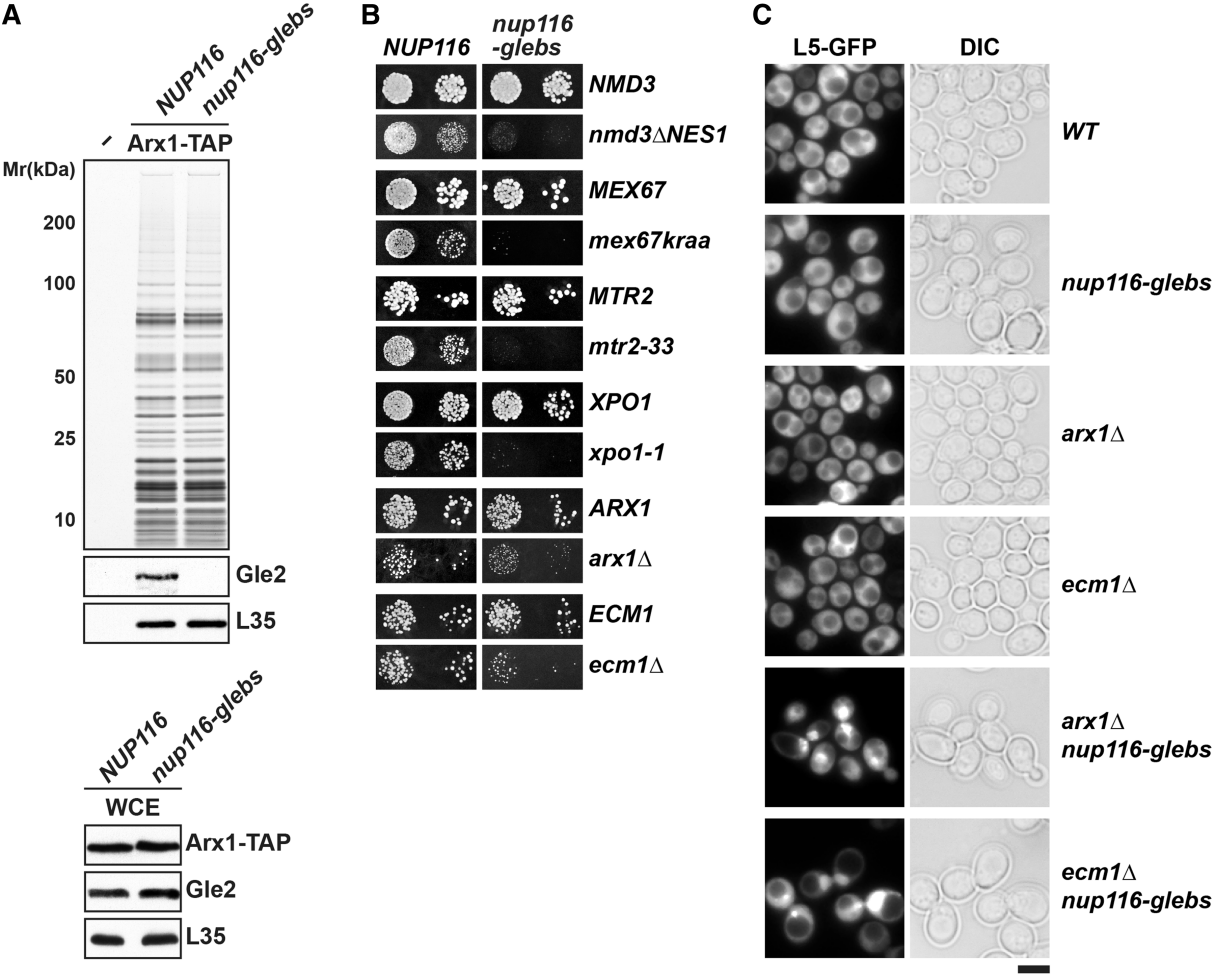
A role for the Nup116-GLEBS domain in pre-60S subunit export. (**A**) Top: Gle2 does not co-enrich with export competent pre-60S subunits in mutant strain expressing *nup116-glebs* allele. TAP purifications using Arx1 as bait were performed in strains expressing WT (*NUP116*) and *nup116-glebs* mutant allele. The calmodulin-sepharose eluates were analyzed on NuPAGE 4–12% gradient gels followed by silver staining and western analyses. The large subunit ribosomal protein L35 served as loading control. Bottom: Gle2 protein is expressed at WT level in strain expressing *nup116-glebs* allele. Western analysis of whole-cell extracts (WCE) derived from indicated strains using the depicted antibodies. The large ribosomal subunit protein L35 served as loading control. (**B**) Genetic interaction between Nup116 and pre-60S export factors. The *nup116-glebs* mutant is synthetically lethal when combined with *nmd3ΔNES1*, *mex67kraa, mtr2-33* and *xpo1-1* mutants and synthetically enhanced with combined with *arx1Δ* and *ecm1Δ*. Strains carrying the WT and mutant alleles were spotted in 10-fold serial dilutions on 5-FOA (SD) plates and grown at 25–30°C for 3–5 days. (**C**) The *arx1Δnup116-glebs* and *ecm1Δnup116-glebs* strains are impaired in pre-60S subunit export. The indicated strains containing L5-GFP reporter were grown at 30°C till mid log phase. Localization of L5-GFP reporter was analyzed by fluorescence microscopy. Scale bar = 5 µm.

The aforementioned biochemical data raised the possibility whether the recruitment of Gle2 to pre-60S subunits requires prior anchoring to the GLEBS domain of Nup116. To further investigate this, we constructed the *nup116-glebs* mutant by mutating the acidic residues E154 and E155 to basic residues. The mutations of these conserved acidic residues in Nup116 to basic residues were reported to impair association with Gle2 (29). We isolated Arx1-TAP from the *nup116-glebs* mutant and analyzed the association of Gle2 by western analysis. Consistent with the idea that Gle2 recruitment to pre-60S subunits required prior anchoring to Nup116, we found that Gle2 was not recruited to pre-60S subunits in the *nup116-glebs* mutant (Figure 7A, top). This was not due a destabilized Gle2 in the *nup116-glebs* mutant, as levels of Gle2 remain unaltered in the *nup116-glebs* mutant as compared with the WT strain (Figure 7A, bottom). Next, using the nup116-glebs allele, we tested whether Nup116 genetically interacts with pre-60S export factors. This was the case. Like the *gle2-gis* mutant, the *nup116-glebs* mutant was either synthetically lethal (*nmd3ΔNES1*, *mex67kraa*, *mtr2-33* and *xpo1-1*) or synthetically enhanced (*arx1Δ* and *ecm1Δ*) when combined with the pre-60S subunit export mutants (Figure 7B). Moreover, the synthetically enhanced *arx1Δnup116-glebs* and *ecm1Δnup116-glebs* double mutant strains exhibit a strong nucleoplasmic accumulation of the large subunit reporter L5-GFP, indicating impairment in pre-60S subunit nuclear export (Figure 7C). Altogether, these data show that Gle2’s GLEBS interacting surface contributes to its recruitment to pre-60S subunits and their nuclear export.

### Nuclear export of mRNAs under stress conditions remains unaffected in the *gle2-patch* mutant

**Figure 8.**
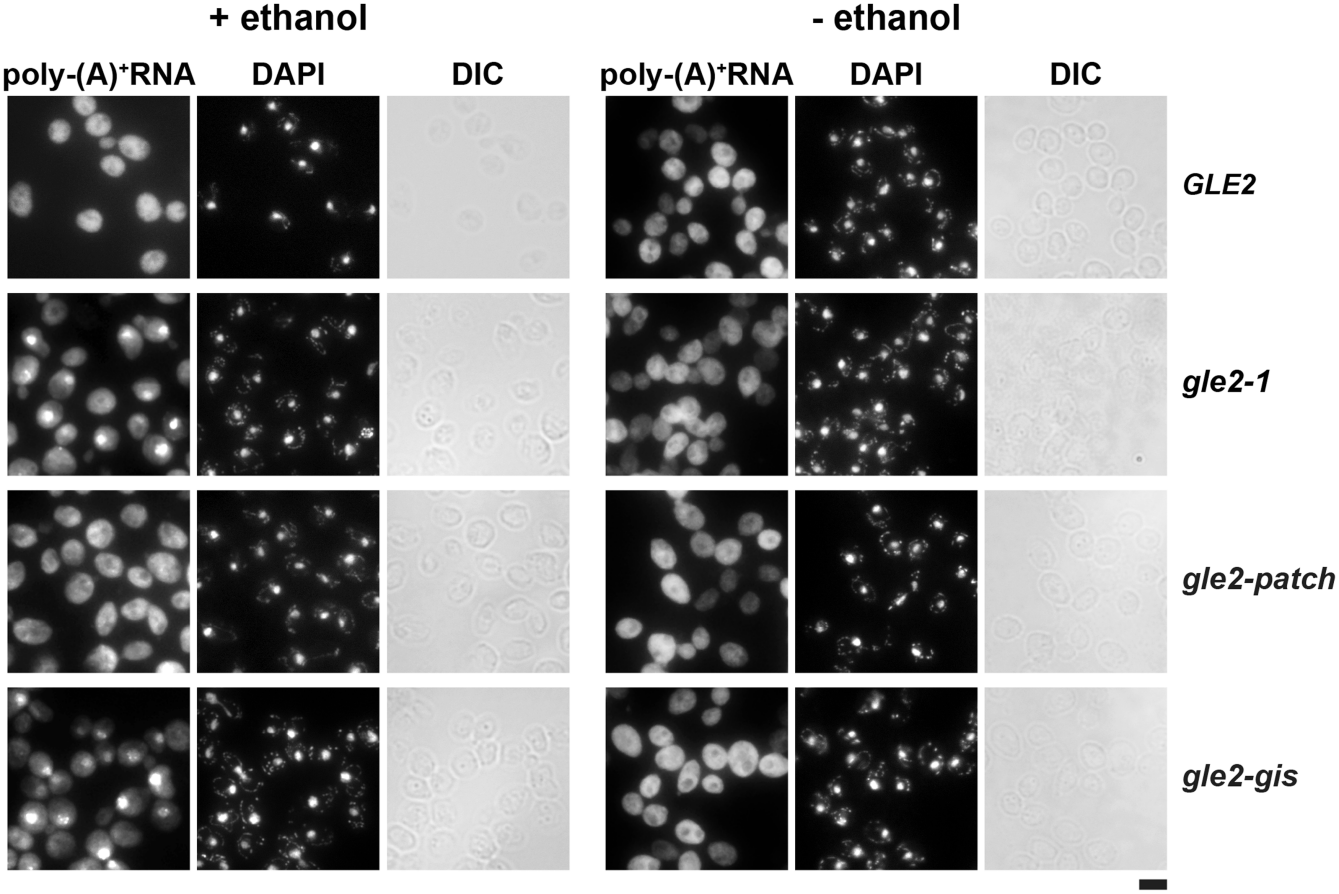
The gle2-patch mutant is able to transport mRNAs to the cytoplasm under mild ethanol stress. The *gle2-1* and *gle2-gis* strains, but not the *gle2-patch* mutant strain, are impaired in poly-(A)^+^ RNA export after ethanol shock. Indicated strains were grown at 30°C till mid log phase and treated with 5% ethanol (v/v) for 5 min (left). Strains without ethanol treatment served as negative controls (right). Localization of poly-(A)^+^ RNA in the indicated strains was assessed by fluorescence *in situ* hybridization using a Cy3-oligo(dT)^30^ probe. Nuclear and mitochondrial DNA was stained with DAPI. Scale bar = 5 µm.

Previously, *gle2Δ* cells were shown to accumulate poly-(A)^+^ RNA in the nucleus on exposure to stress conditions such as heat shock or treatment with ethanol or benzyl alcohol (31). These data prompted us to examine the contribution of the GLEBS-interacting surface and basic patch of Gle2 to the nuclear export of poly-(A)^+^ RNA on exposure to ethanol stress (5% v/v, 5 min). In agreement with previous observations, we found that the *gle2-1* mutant displayed a strong nuclear accumulation of poly-(A)^+^ RNA at 30°C in >95% of cells on exposure to mild ethanol stress (Figure 8). In support of our hypothesis that the GLEBS-interacting surface of Gle2 contributes to the nuclear export of mRNAs under stress conditions (24), we found that the *gle2-gis* mutant showed a strong nuclear accumulation of poly-(A)^+^ RNA at 30°C under these conditions (Figure 8). Localization of both ribosomal subunit reporters (L5-GFP and S2-GFP) and NPC distribution (Nup49-GFP) remained unaltered in the *gle2-gis* strain under the same conditions (Supplementary Figure S2A and B). These data suggest a specific requirement of the GLEBS-interacting surface in the export of mRNAs under mild ethanol stress conditions. In contrast, no nuclear accumulation of poly-(A)^+^ RNA was observed when the *gle2-patch* mutant was exposed to ethanol stress (Figure 8). Also, localization of ribosomal subunit reporters (L5-GFP and S2-GFP) and NPC distribution was not affected in this strain when exposed to ethanol stress (Supplementary Figure S2B). Thus, although the *gle2-patch* mutant is unable to function in pre-60S subunit export, it is competent to transport mRNAs to the cytoplasm under stress conditions.

## DISCUSSION

Eukaryotic cells need to rapidly export assembled pre-ribosomal particles, which are among the largest and most abundant cargoes that need to cross the permeability barrier of the NPC. An actively growing yeast cell is estimated to transport 25 pre-ribosomal particles per minute per NPC (64). Such a massive efflux requires an efficient active transport machinery to translocate pre-ribosomal subunits through the NPC transport channel. Transport of large cargos may be hindered during passage through the NPCs; pre-60S subunits therefore rely on multiple receptors for their efficient translocation (16,65). Genetic approaches in budding yeast have uncovered a handful of factors that directly mediate efficient pre-60S subunit nuclear export. All export factors identified to date use the FG-interaction meshwork to translocate pre-ribosomal subunits through the active channels of NPCs. Here, we reveal an adjunct unrecognized role for the non-FG interacting mRNA export factor Gle2 in pre-60S subunit export.

First, we found that Gle2 genetically interacts with pre-60S export factors (Xpo1, Nmd3, Bud20, Arx1, Ecm1, Npl3 and Mex67-Mtr2), but not with factors required for early pre-60S subunit assembly (Nog1 and Nop7) (Figure 1), suggesting that Gle2 functions in pre-60S subunit export. Second, in the synthetically enhanced *arx1Δgle2-1* and *ecm1Δgle2-1* mutant strains, nuclear export of pre-60S subunits was strongly impaired (Figure 2A). In contrast, export of pre-40S subunits and mRNA was not affected in these double mutants (Supplementary Figure S1B), indicating a specific impairment in pre-60S subunit nuclear export. Third, the presence of halfmers in the polysome profile of the *gle2-1* mutant, again points to a deficit of cytoplasmic 60S subunits (Figure 2A). Finally, using TAP, we showed that Gle2 was recruited to late pre-60S particles loaded with the export factors Nmd3, Bud20 and Mex67-Mtr2 (Figure 3A). Together, these data support the notion that Gle2 functions in the nuclear export of the large pre-ribosomal subunit.

Structure-guided functional studies identified two conserved surfaces used by Gle2 to function in pre-60S subunit export. The first interaction surface is a conserved basic patch present along the circumference of the β-propeller fold of Gle2 required for pre-60S subunit binding (Figures 4A and 6A). This basic patch was not required to anchor Gle2 to the NPC, as Nup116-TAP efficiently co-enriched the *gle2-patch* mutant (Figure 6B). Whether this basic patch binds to an exposed rRNA and/or a negatively charged protein factor(s) to recruit Gle2 to late pre-60S ribosomes remains to be determined. A second surface required for Gle2 function in pre-60S export is the GLEBS-interacting surface that anchors Gle2 to the GLEBS motif of Nup116 *via* non-FG interactions. Notably, biochemical studies showed that mutations in the GLEBS-interacting surface of Gle2 compromised its recruitment to Nup116 as well as to pre-60S subunits (Figure 6A and B). Further, mutations in the GLEBS domain of Nup116 also abolished the recruitment of Gle2 to late pre-60S subunits (Figure 7A). Together, these data suggest that prior anchoring to Nup116 is important to recruit Gle2 to pre-60S subunits.

Based on all the data, we propose the following model: Gle2 uses a distinct interaction platform to bind an export competent pre-60S subunit and the interaction between Gle2, bound to the pre-60S subunit and GLEBS-motif of Nup116 at the NPC aids the transit of the bound cargo through the transport channel. Studies primarily in budding yeast have aided the identification of essential factors (Xpo1, Nmd3, Mex67-Mtr2) and several auxiliary factors (Npl3, Arx1, Ecm1, Bud20), which translocate pre-60S subunits through the NPC. Whether all factors decorate each pre-60S subunit for export remains unclear. One possibility could be that not all the export factors are present on the pre-60S subunit at the same time; rather, a minimal complement of export receptors may ensure rapid transport to the cytoplasm. A rapid transit through the NPC is especially important, as any delay within the channel may hinder transport of other cargoes. Therefore, it is conceivable that several export factors, including interactions within the NPC channel, are required to guarantee efficient exit. Gle2 may play a crucial role in the nuclear export of the pre-60S subunit within the transport channel, especially if the pre-60S subunit fails to recruit its full complement of export factors. One attractive hypothesis could be that by lining the NPC channel *via* its interaction with the GLEBS-motif of Nup116, Gle2 may prevent kinetic delays experienced by export cargos during translocation, in case pre-60S subunits failed to recruit their full complement of transport receptors. Thus, pre-ribosomal particles, and potentially mRNPs, may sample both FG-repeats as well as distinct Gle2 surfaces to quickly traverse the NPC.

How does Gle2 function in the mRNA export pathway? Consistent with previous observations, we found that Gle2 becomes crucial for the nuclear export of mRNAs under stress conditions. Furthermore, our cell-biological studies revealed that the GLEBS interacting surface of Gle2, but not the basic patch, contributes to export mRNAs under stress conditions (Figure 8). The precise interaction surface of Gle2 that contributes to nuclear export of mRNAs under stress remains to be determined. Here, structure-guided functional analyses aided the design of the *gle2-patch* allele that specifically contributes to pre-60S subunit nuclear export. A similar approach may be used to determine the interaction surface used by Gle2 to function in the nuclear export of mRNAs under stress conditions.

The toroid β-propeller fold of WD40 domain protein provides an ideal interaction platform to aid the assembly and regulation of various complexes (66). Structural analyses of protein complexes containing this domain have revealed that the majority of the interacting proteins/peptides bind on the top of the β-propeller, although several also bind to the circumference as well as the bottom. The versatility of the WD40 fold of Rae1/Gle2 is reflected in its involvement in diverse processes such as mRNA export, spindle assembly, chromosome segregation and protein turnover. Although Rae1 was shown to interact with a nuclear mitotic apparatus *via* a GLEBS-like motif, the details of its interaction with the cohesion Smc1 remain unclear. Moreover, how Rae1 interacts with the Cdh1-activated anaphase-promoting complex and the HiW ubiquitin ligase to prevent aneuploidy and regulate synaptic plasticity, respectively, remains unknown. Interaction with one partner may hinder interactions with other binding partners, potentially to further regulate Rae1/Gle2 function *in vivo*. Revealing the interaction network, as well as uncovering the surfaces used by Rae1/Gle2 to engage with multiple functional partners, will help elucidate the mechanisms by which WD40 domains have evolved to co-ordinate distinct biological pathways.

## SUPPLEMENTARY DATA

Supplementary Data are available at NAR Online, including [67].

## FUNDING

Swiss National Science Foundation (SNSF) and the ETH Zurich (to V.G.P.); Starting Grant Award [EURIBIO260676 to V.G.P.] from the European Research Council. Funding for open access charge: SNSF and the European Research Council.

*Conflict of interest statement*. None declared.

## Supplementary Material

Supplementary Data
