# Supplementary material for: Non-FG mediated transport of the large pre-ribosomal subunit through the nuclear pore complex by the mRNA export factor Gle2
Source: Nucleic Acids Res. 2013 Jul 31;41(17):8266–79. doi: 10.1093/nar/gkt675 (PMC3783196; doi:10.1093/nar/gkt675)
Supplement: Supplementary Data [file supp_41_17_8266__index.html]

Non-FG mediated transport of the large pre-ribosomal subunit through the nuclear pore complex by the mRNA export factor Gle2 — Non-FG mediated transport of the large pre-ribosomal subunit through the nuclear pore complex by the mRNA export factor Gle2 — Supplementary Data 

# Non-FG mediated transport of the large pre-ribosomal subunit through the nuclear pore complex by the mRNA export factor Gle2

## 

files

**Files in this Data Supplement:**

- Supplementary Data - pdf file
