# Supplementary material for: Non-FG mediated transport of the large pre-ribosomal subunit through the nuclear pore complex by the mRNA export factor Gle2
Source: Nucleic Acids Res. 2013 Jul 31;41(17):8266–79. doi: 10.1093/nar/gkt675 (PMC3783196; doi:10.1093/nar/gkt675)
Supplement: Supplementary Data [file supp_gkt675_nar-01029-v-2013-File010.pdf]

Supplementary Data

Non-FG mediated transport of the large pre-ribosomal subunit through the nuclear pore complex by the mRNA export factor Gle2

Laura Occhipinti, Yiming Chang, Martin Altvater, Anna Maria Menet, Stefan Kemmler and Vikram Govind Panse

Content:

Supplementary Figure S1: NPC distribution and localization of pre-40S subunits and mRNA remain unaltered in *gle2* mutant strains.

Supplementary Figure S2: Localization of pre-ribosomal subunits and NPC distribution remain unaltered in *gle2-1*, *gle2-patch* and *gle2-gis* mutant strains under mild ethanol stress.

Supplementary Table S1: Yeast strains used in this study.

Supplementary Table S2: Plasmids used in this study.

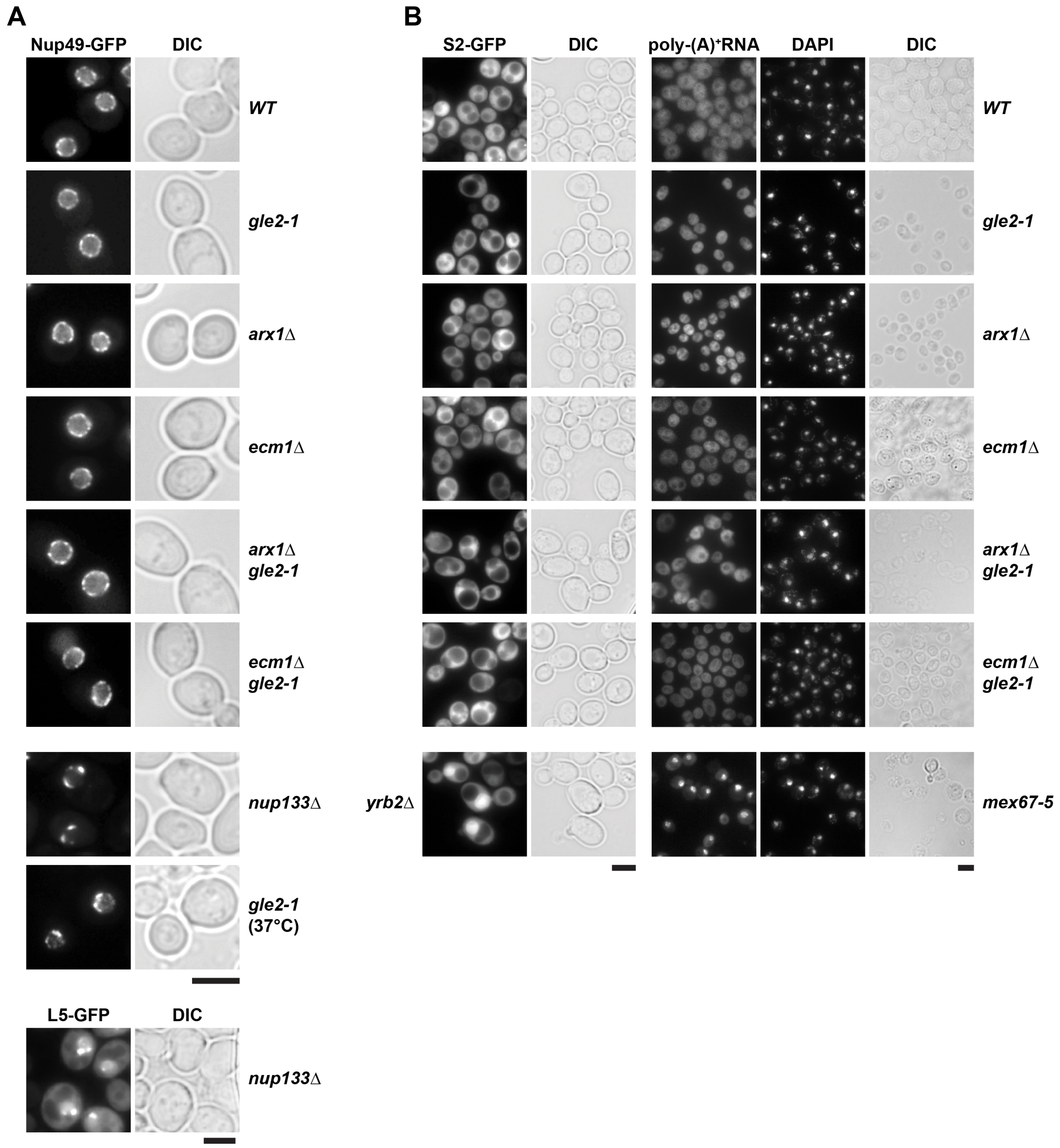

Supplementary Figure S1: NPC distribution and localization of pre-40S subunits and mRNA remain unaltered in *gle2* mutant strains. (A) Indicated strains containing Nup49-GFP were grown at 30°C till mid log phase, and localization of Nup49-GFP was analyzed by fluorescence microscopy. The *gle2-1* (shifted to 37°C for 5 h) and *nup133*Δ (at 30°C) mutants served as positive controls for “pore-clustering” phenotype. To show nuclear accumulation of pre-60S subunits in the “pore-clustering” *nup133*Δ strain, cells containing L5-GFP reporter were grown at 30°C till mid log phase and analyzed by fluorescence microscopy. (B) Indicated strains containing S2-GFP reporters (left) or no reporter (right) were grown at 30°C till mid log phase. Localization of S2-GFP was monitored by fluorescence microscopy.

The *yrb2*Δ strain served as positive control for impaired nuclear export of pre-40S subunits. Localization of poly-(A)$^+$ RNA in the indicated strains was assessed by FISH using a Cy3-oligo(dT)^{30} probe. Nuclear and mitochondrial DNA was stained with DAPI. The *mex67-5* mutant, grown at 30°C till mid log phase and shifted to 37°C for 1 h, served as positive control for impaired export of poly-(A)$^+$ RNA. Scale bar = 5 µm.

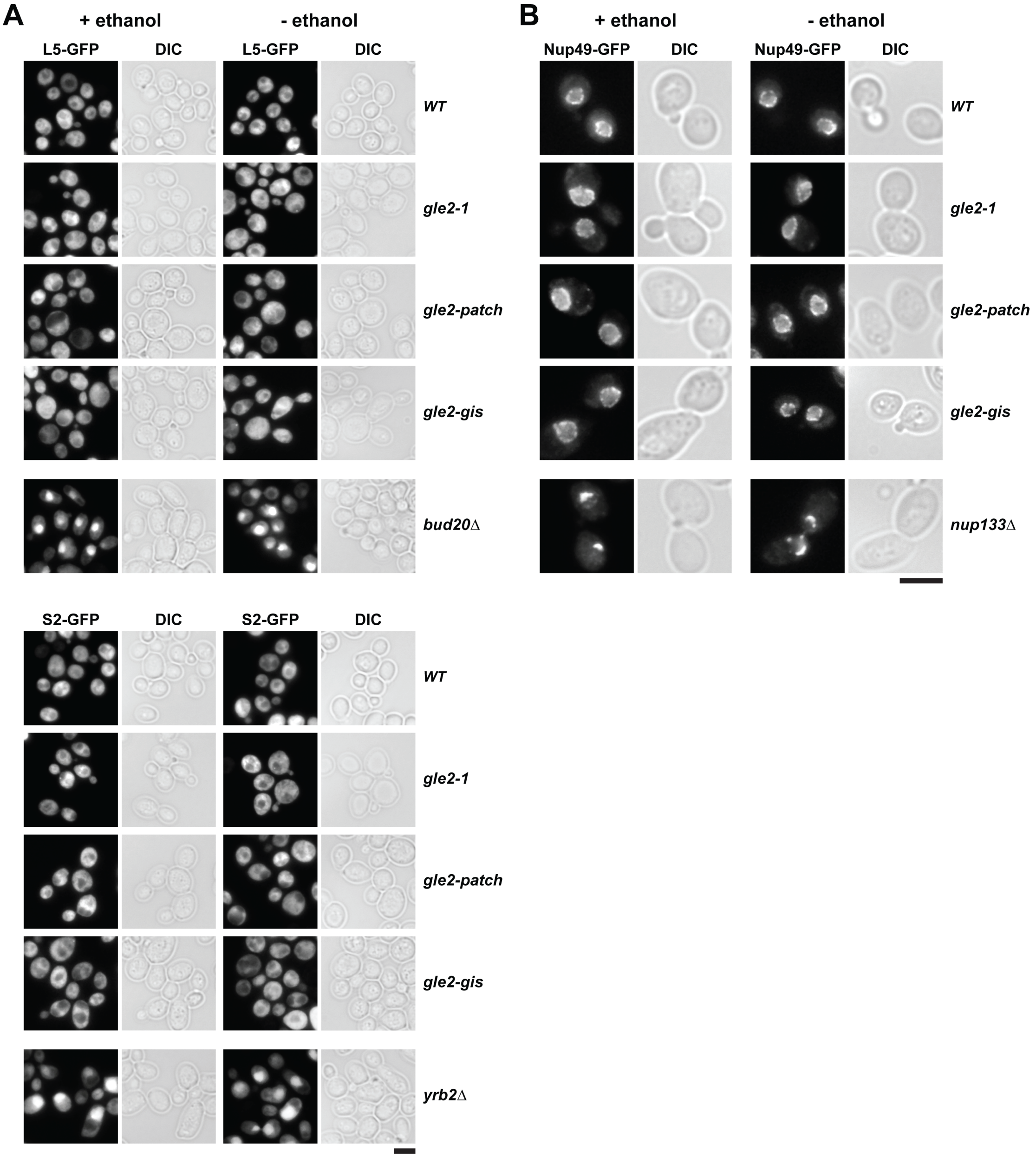

Supplementary Figure S2: Localization of pre-ribosomal subunits and NPC distribution remain unaltered in *gle2-1*, *gle2-patch* and *gle2-gis* mutant strains under mild ethanol stress. The indicated strains containing L5-GFP or S2-GFP reporter (A) or Nup49-GFP (B) were grown at 30°C till mid log phase and treated with or without 5% ethanol (v/v) for 5 min. After fixation with 4% formaldehyde (w/v), localization of GFP proteins was analyzed by fluorescence microscopy. *bud20*Δ (for impaired pre-60S export), *yrb2*Δ (for impaired pre-40S export) and *nup133*Δ mutant strains (for "pore-clustering" phenotype) served as positive controls. Scale bar = 5 µm.

Supplementary Table S1: Yeast strains used in this study.

Strain	Genotype	Source
BY4741	*MATa ura3 his3 leu2 met15 TRP1*	Open Biosystems
gle2-1	*MATα gle2-1 ade2-1 ADE3 ura3-1 his3-11,15 leu2-3,112 trpl-1 LYS2 canl-100*	(24)
xpo1 shuffle *gle2-1*	*MATα ura3 his3 leu2 xpo1::KANMX6 gle2-1 <pRS316-XPO1>*	This study
mtr2 shuffle *gle2-1*	*MATα ura3 leu2 trp1 mtr2::HIS3MX gle2-1 <pRS316-MTR2>*	This study
mex67 shuffle *gle2-1*	*MATα ura3 leu2 trp1 mex67::HIS3MX gle2-1 <pRS316-MEX67>*	This study
bud20Δ gle2-1	*MATα gle2-1 ura3 his3 leu3 trp1 bud20::NATMX4*	This study
ecm1Δ gle2-1	*MATa ura3 his3 leu2 ECM1::KANMX6 gle2-1*	This study
arx1Δ gle2-1	*MATa ura3 his3 leu2 trp1 ARX1::KANMX6 gle2-1*	This study
nog1 shuffle *gle2-1*	*MATa ura3 his3 leu2 nog1::KANMX6 gle2-1 <pRS316-NOG1>*	This study
nop7 shuffle *gle2-1*	*MATa ura3 his3 leu2 trp1 nop7::KANMX6 gle2-1 <pRS316-NOP7>*	This study
Ssf1-TAP	*MATα his3 leu2 ura3 SSF1-TAP::TRP1*	(54)
Rix1-TAP	*MATα ura3 his3 leu2 trp1 RIX1-TAP::TRP1*	(54)
Arx1-TAP	*MATα ura3 his3 leu2 ARX1-TAP::TRP1*	(54)
Nmd3-TAP	*MATa his3 leu2 ura3 NMD3-TAP::HIS3MX6*	Open Biosystems
Kre35-TAP	*MATα ura3 his3 leu2 trp1 KRE35-TAP::TRP1*	(54)
Arx1-TAP *gle2Δ*	*MATa his3 leu2 ura3 Arx1-TAP::KANMX6 gle2:: NATMX4*	This study
nup116Δ	*MATa his3 leu2 trp1 ura3 nup116::HIS3*	(27)
Arx1-TAP *nup116Δ*	*MATa his3 leu2 ura3 nup116::HIS3 ARX1-TAP::TRP*	This study

Nup100-TAP	*MATa his3 leu2 ura3 Nup100-TAP::HIS3MX6*	Open Biosystems
Nup116-TAP	*MATα his3 leu2 ura3 Nup116-TAP::HIS3MX6*	(27)
Nup116-TAP *gle2Δ*	*MATα his3 leu2 ura3 Nup116-TAP::HIS3MX6 gle2::NATMX4*	This study
arx1Δ	*MATa ura3 his3 leu2 arx1::KANMX6*	Open Biosystems
ecm1Δ	*MATa ura3 his3 leu2 met15 ecm1::KANMX6*	Open Biosystems
nmd3 shuffle *gle2-1*	*MATα ura3 leu2 trp1 nmd3::HIS3MX gle2-1 <pRS316-NMD3>*	This study
npl3Δ gle2-1	*MATα gle2-1 ura3-1 his3 leu2, trp1 npl3::KANMX6*	This study
yrb2Δ	*MATa his3 leu2 ura3 yrb2::KANMX6*	Open Biosystems
mex67 shuffle	*MATα ura3 leu2 trp1 mex67::HIS3MX <pRS316-MEX67>*	This study
nup133Δ	*MATa his3 leu2 ura3 nup133::KANMX6*	Open Biosystems
gle2Δ	*MATa ura3 his3 leu2 gle2::KANMX6*	This study
mex67 shuffle *nup116Δ*	*MATα ura3 leu2 trp1 nup116::NATMX4 mex67::HIS3MX <pRS316-MEX67>*	This study
mtr2 shuffle *nup116Δ*	*MATa ura3 leu2 trp1 nup116::NATMX4 mtr2::HIS3MX <pRS316-MTR2>*	This study
nmd3 shuffle *nup116Δ*	*MATa ura3 leu2 trp1 nup116::NATMX4 nmd3::HIS3MX <pRS316-NMD3>*	This study
xpo1 shuffle *nup116Δ*	*MATa ura3 leu2 his3 nup116::NATMX4 xpo1::KANMX6 <pRS316-XPO1>*	This study
Pgal-NUP116 arx1Δ	*MATa ura3 his3 leu2 met15 arx1::KANMX6 Pgal-NUP116::NATMX4*	This study
Pgal-NUP116 ecm1Δ	*MATa ura3 his3 leu2 met15 ecm1::KANMX6 Pgal-NUP116::NATMX4*	This study

Supplementary Table S2: Plasmids used in this study.

Plasmid	Relevant markers	Source
pRS314-*NMD3*	*NMD3 CEN TRP1*	(54)
pRS314-*nmd3ΔNES1*	*nmd3ΔNES1 CEN TRP1*	(54)
pRS315-*XPO1*	*XPO1 CEN LEU2*	(12)
pRS315-*xpo1-1*	*xpo1-1 CEN LEU2*	(12)
pRS314-*MTR2*	*MTR2 CEN TRP1*	(54)
pRS314-*mtr2-33*	*mtr2-33 CEN TRP1*	(54)
pUN100-*MEX67*	*MEX67 CEN TRP1*	(54)
pRS315-*mex67kraa*	*mex67kraa CEN TRP1*	(54)
pRS315-*mex67-5*	*mex67-5 CEN LEU2*	(12)
pRS313-*ECM1*	*ECM1 CEN HIS3*	This study
pRS313-*ARX1*	*ARX1 CEN HIS3*	This study
pRS315-*NOP7*	*NOP7 CEN LEU2*	This study
pRS315-*nop7-1*	*nop7-1 CEN LEU2*	(51)
pRS315-*nog1*	*NOG1 CEN LEU2*	This study
pRS315-*nog1-11*	*nog1-11 CEN LEU2*	(52)
pRS315-*gle2-R186E,K305E*	*gle2-R186E,K305E CEN LEU2*	This study
pRS315-*gle2-R168E,R212E*	*gle2-R168E,R212E CEN LEU2*	This study
pRS313-*GLE2*	*GLE2 CEN HIS3*	This study
pRS314-*GLE2*	*GLE2 CEN TRP1*	This study
pRS315-*GLE2*	*GLE2 CEN LEU2*	(24)
pRS316-*GLE2*	*GLE2 CEN URA3*	This study
pRS315-*NUP116*	*NUP116 CEN LEU2*	This study
pRS315-*nup116-E154,155R*	*nup116-E154,155R CEN LEU2*	This study
pUN100-*NUP49-GFP*	*NUP49-GFP CEN LEU2*	This study

pRS316-*NUP116*	*NUP116 CEN URA3*	This study
pRS315-S2-eGFP	*RPS2-eGFP CEN LEU2*	(18)
pRS315-L5-eGFP	*RPL5-eGFP CEN LEU2*	(18)
pRS316-S2-eGFP	*RPS2-eGFP CEN URA3*	(67)
pRS316-L5-eGFP	*RPL5-eGFP CEN URA3*	(18)

References for Supplementary Data

67. Milkereit, P., Strauss, D., Bassler, J., Gadal, O., Kühn, H., Schütz, S., Gas, N., Lechner, J., Hurt, E. and Tschochner, H. (2003) A Noc complex specifically involved in the formation and nuclear export of ribosomal 40 S subunits. *J. Biol. Chem.*, **278**, 4072-4081.
